# Prox1 represses IL-2 gene expression by interacting with NFAT2

**DOI:** 10.18632/oncotarget.17278

**Published:** 2017-04-20

**Authors:** Shujie Zhang, Ning Yu, Linfang Wang, Yanfeng Liu, Yuying Kong, Jing Liu, Youhua Xie

**Affiliations:** ^1^ Eye Institute, Eye and ENT Hospital, Shanghai Medical College, Fudan University, Shanghai 200031, China; ^2^ Department of Dermatology, Shanghai Skin Disease Hospital, Shanghai 200050, China; ^3^ Key Laboratory of Medical Molecular Virology (MOE and MOH), Institute of Biomedical Sciences, Shanghai Medical College, Fudan University, Shanghai 200032, China

**Keywords:** gene regulation, prospero-related homeobox 1, repression, nuclear factor of activated T cells 2

## Abstract

Interleukin-2 (IL-2) is critical for T lymphocyte activation and regulated by many transcriptional factors. Prospero-related homeobox 1 (Prox1) is a multifunctional transcription factor, which can work as either a transcriptional activator or repressor depending on the cellular and developmental environment. We previously reported the Prox1 expression in T cells, raising the possibility of Prox1 involvement in the regulation of T cell function and IL-2 production. Here we demonstrated that the Prox1 expression in CD4+ T cells was downregulated by T cell receptor (TCR) activation. Overexpression of Prox1 attenuated IL-2 production, while knockdown of endogenous Prox1 by small interfering RNA increased IL-2 expression. Mechanistically, we showed that Prox1 inhibited the IL-2 promoter activity, and associated with the minimal IL-2 promoter. Prox1 repressed the nuclear factor of activated T cells 2 (NFAT2)-dependent transactivation of IL-2 gene by physically binding to NFAT2. The N-terminal region of Prox1 was essential for the binding and repression. In summary, our findings established Prox1 as a negative regulator in IL-2 gene expression through the direct interaction with NFAT2.

## INTRODUCTION

T cell activation results into the secretion of interleukin-2 (IL-2) [1]. IL-2 crucially affects the activation and differentiation of T cells in an autocrine or paracrine manner. IL-2 also plays an important role in the activation-induced cell death (AICD) and Treg function [2]. However, the regulatory mechanisms of initiation, maintenance and suppression of IL-2 gene expression remain elusive.

IL-2 expression regulation occurs at transcriptional and post-transcriptional levels [3]. IL-2 mRNA transcription is regulated mostly within the 300 bp upstream of the transcription start site. Several positive transcription factors including nuclear factor-κB (NF-κB), activated protein-1 (AP-1) and nuclear factor of activated T cells (NFAT) bind to this region [2]. NFAT molecules act synergistically with several co-regulators, resulting in synergistic activation, e.g. AP-1 and CBP/p300, or repression, e.g. Foxp3, ICER and peroxisome proliferator-activated receptor γ (PPARγ) of IL-2 transcription [4–8].

Prox1 function was associated with the developmental process of lymphatic vasculature [9, 10] and liver [11], and regulation of skeletal muscle phenotype [12]. Our lab has previously performed a series of experiments to investigate the function of Prox1. Prox1 acted as a co-repressor of liver receptor homologue 1 (LRH1) to suppress the activation of cholesterol 7α-hydroxylase (CYP7A1), the key gene in bile acid synthesis [13]. More recently, we have shown that Prox1 directly recruited the lysine-specific demethylase 1 (LSD1)/nucleosome remodeling and histone deacetylase (NuRD) complex to epigenetically co-repress CYP7A1 transcription [14]. We also proved that Prox1 inhibited replication of hepatitis B virus [15]. In regard to carcinogenesis, Prox1 increased the expression of β-catenin, thus enhancing the proliferation of hepatocellular carcinoma [16]. Furthermore, Prox1 upregulated expression of hypoxia-inducible factor-1α (HIF-1α), thus promoting hepatocellular carcinoma metastasis [17]. However, the role for Prox1 in the immune regulation has yet to be elucidated.

In our previous study, we demonstrated that Prox1 was expressed in Jurkat cells and primary human CD4^+^ T cells [18]. Prox1 downregulated expression of IFN-γ by suppressing the promoter activity [18]. Considering the essential role of IL-2 in T cell activation, here we sought to investigate whether and how Prox1 regulates the IL-2 expression.

## RESULTS

### Prox1 expression is decreased during T cell activation

**Figure 1:**
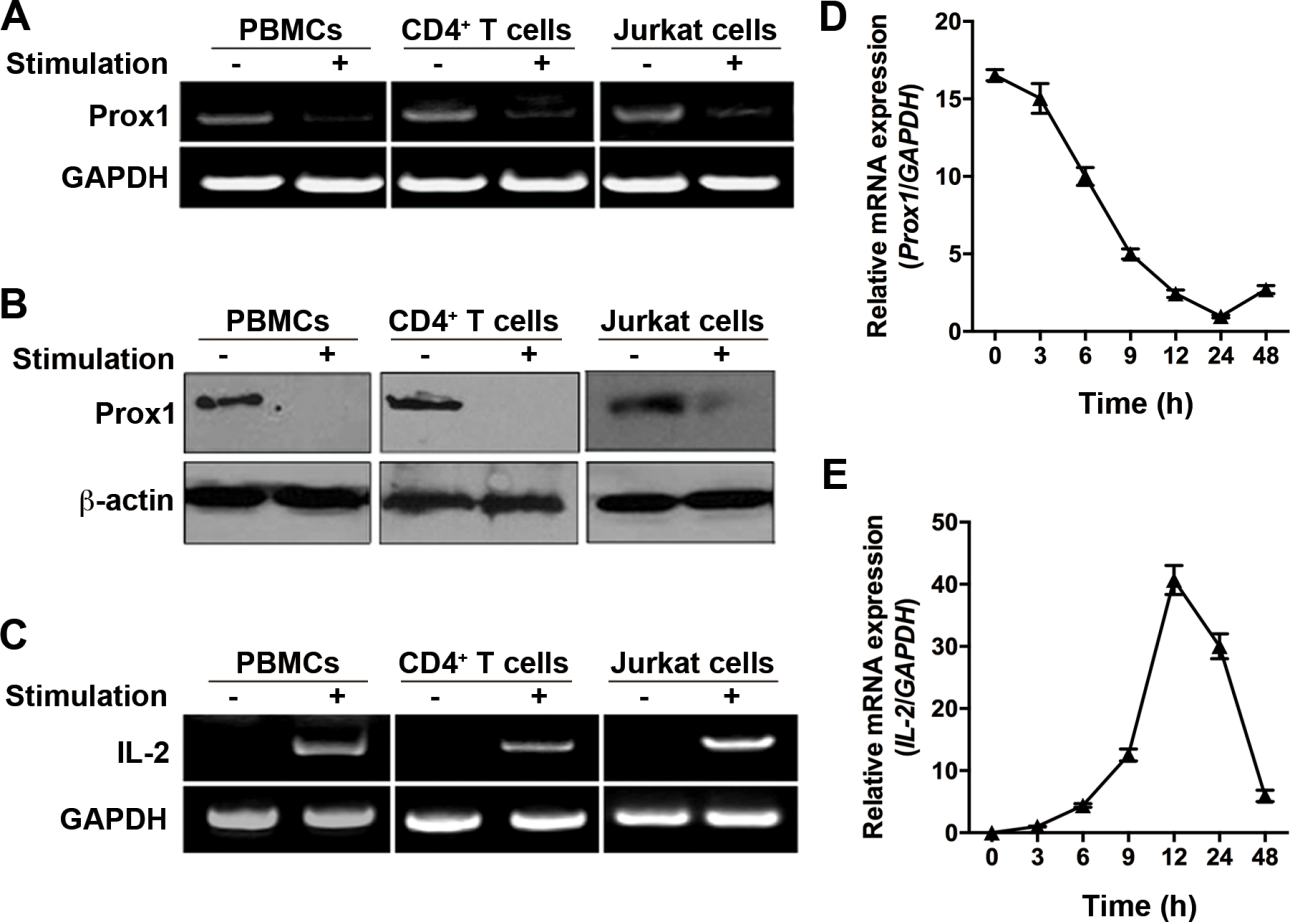
Expression of Prox1 mRNA and protein in T cells (**A, B** and **C**) PBMCs, naïve CD4^+^ T cells and Jurkat cells were isolated, and stimulated with PHA and anti-CD3/CD28 Dynabeads for 24 h respectively. (A) Prox1 and (C) IL-2 mRNA levels were measured by RT-PCR, while (B) Prox1 protein levels were assessed by Western blot. For (A), (B) and (C), the data represent one of three independent experiments. (**D** and **E**) Naïve CD4^+^ T cells were stimulated with anti-CD3/CD28 Dynabeads for indicated times. (D) Prox1 and (E) IL-2 mRNA levels were measured by real-time PCR. Gene expression is normalized against the amount of GAPDH mRNA. The data from represent the mean ± SEM of three experiments.

First, we investigated Prox1 expression in human PBMCs, isolated naïve CD4^+^ T cells and cultured Jurkat cells, under basal and stimulated states. The results demonstrated that Prox1 mRNA and protein was constitutively expressed in unstimulated PBMCs, naïve CD4^+^ T cells as well as Jurkat cells (Figure 1A, 1B, and Supplementary Figure 1). Stimulation of PBMCs with PHA, and stimulation of T cells with anti-CD3/CD28 Dynabeads for 24 h induced a precipitous drop in Prox1 mRNA and protein level (Figure 1A and 1B). In parallel, a substantial increase was observed in the level of IL-2 mRNA following the stimulation (Figure 1C). Moreover, we activated the naïve CD4^+^ T cells, and examined the kinetics of Prox1 and IL-2 mRNA expression. The Prox1 level declined to its lowest point at 24 h (Figure 1D), and this coincided with a sharp increase of IL-2 mRNA level (Figure 1E).

### Overexpression of Prox1 attenuates IL-2 expression

**Figure 2:**
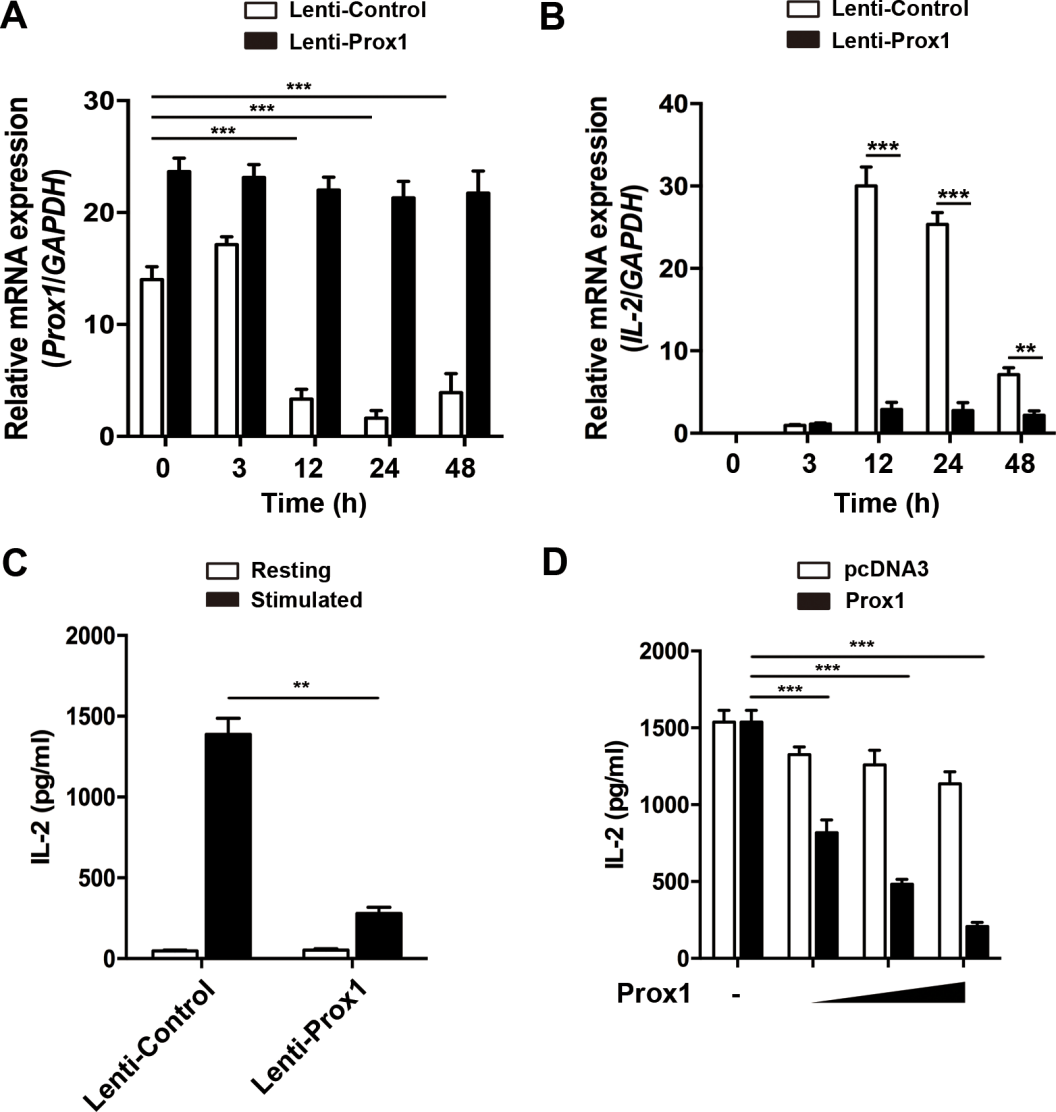
Overexpression of Prox1 inhibits IL-2 expression (**A** and **B**) Lentiviral vector expressing Prox1 gene and GFP, or GFP alone, were generated. Jurkat cells were infected with Prox1 lentivirus or the control lentivirus. The GFP^+^ Jurkat cells were sorted 48 h post-infection, and stimulated with anti-CD3/CD28 Dynabeads for indicated times. (A) Prox1 and (B) IL-2 mRNA levels were assessed by real-time PCR, while (**C**) IL-2 protein levels at 24 h were measured by ELISA. (**D**) Jurkat cells were transiently transfected with increasing amount of Prox1 plasmids (0.125, 0.25 and 0.5 μg/ml) or control vector (pcDNA3), and stimulated with anti-CD3/CD28 Dynabeads for 24 h. The IL-2 protein levels were measured by ELISA. The data from represent the mean ± SEM of three experiments, **P < 0.01, ***P < 0.001, one-way ANOVA (A and D), Student’s *t*-test (B and C).

Considering the negative correlation between Prox1 and IL-2 expression, we speculated that Prox1 may decrease the expression of IL-2. To this end, we determined whether ectopic Prox1 in Jurkat cells could modulate IL-2 expression. Jurkat cells were infected with Prox1 lentivirus or the control lentivirus, and stimulated with anti-CD3/CD28 Dynabeads for times ranging from 0 to 48 h. We observed a sustained expression of Prox1 mRNA in Jurkat cells following the stimulation (Figure 2A). The overexpressed Prox1 suppressed the IL-2 mRNA and protein expression (Figure 2B and 2C). In addition, Jurkat cells were transiently transfected with different doses of Prox1 expression plasmids, and IL-2 secretion was determined 24 h after stimulation of anti-CD3/CD28 Dynabeads. Prox1 suppressed the secretion of IL-2 in a dose-dependent manner (Figure 2D).

### Knockdown of endogenous Prox1 increases IL-2 expression

**Figure 3:**
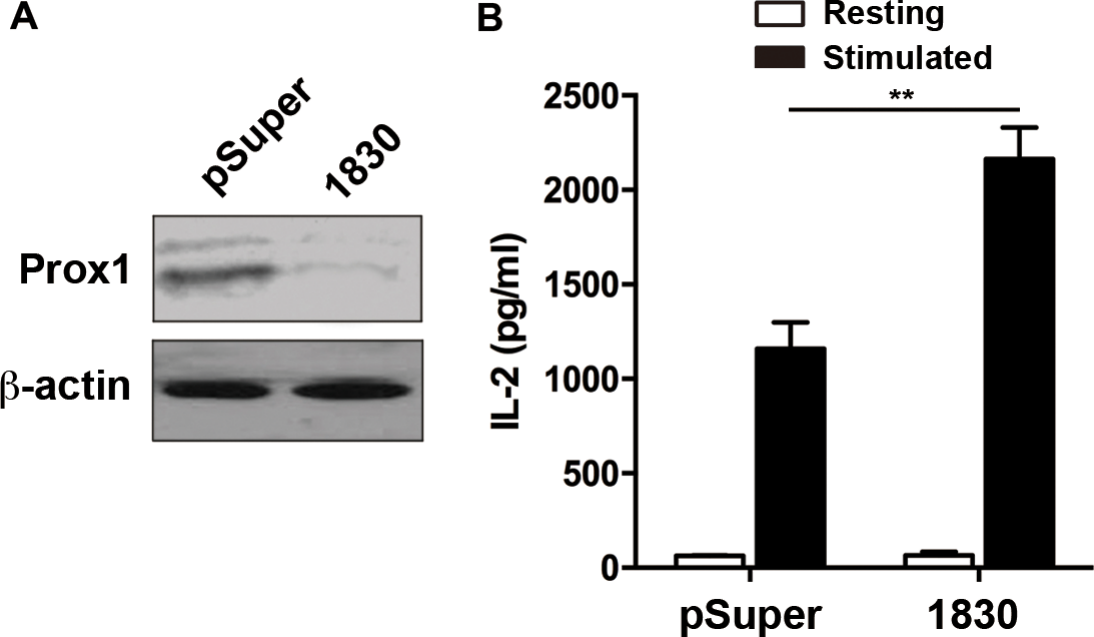
Knockdown of Prox1 increases IL-2 expression Jurkat cells were transfected with either pSuper vector or pSuper-Prox1-1830 (1830), containing GFP. The GFP^+^ Jurkat cells were sorted 36 h post-transfection. (**A**) The protein expression of Prox1 was examined by Western blot. One experiment representative of three experiments. (**B**) The siRNA-transfected Jurkat cells were stimulated with anti-CD3/CD28 Dynabeads for 24 h, and IL-2 production was examined by ELISA. The data from represent the mean ± SEM of three experiments, **P < 0.01, Student’s *t*-test.

To further explore the Prox1-mediated suppression of IL-2 expression, knockdown of Prox1 was performed using plasmids expressing Prox1-targeting siRNA. Western blot revealed that Prox1 expression was markedly reduced in the Jurkat cells transfected with the Prox1-targeting siRNA vector as compared with the control (Figure 3A). Functionally, knockdown of endogenous Prox1 enhanced IL-2 production (Figure 3B).

### Prox1 inhibits IL-2 promoter activity

**Figure 4:**
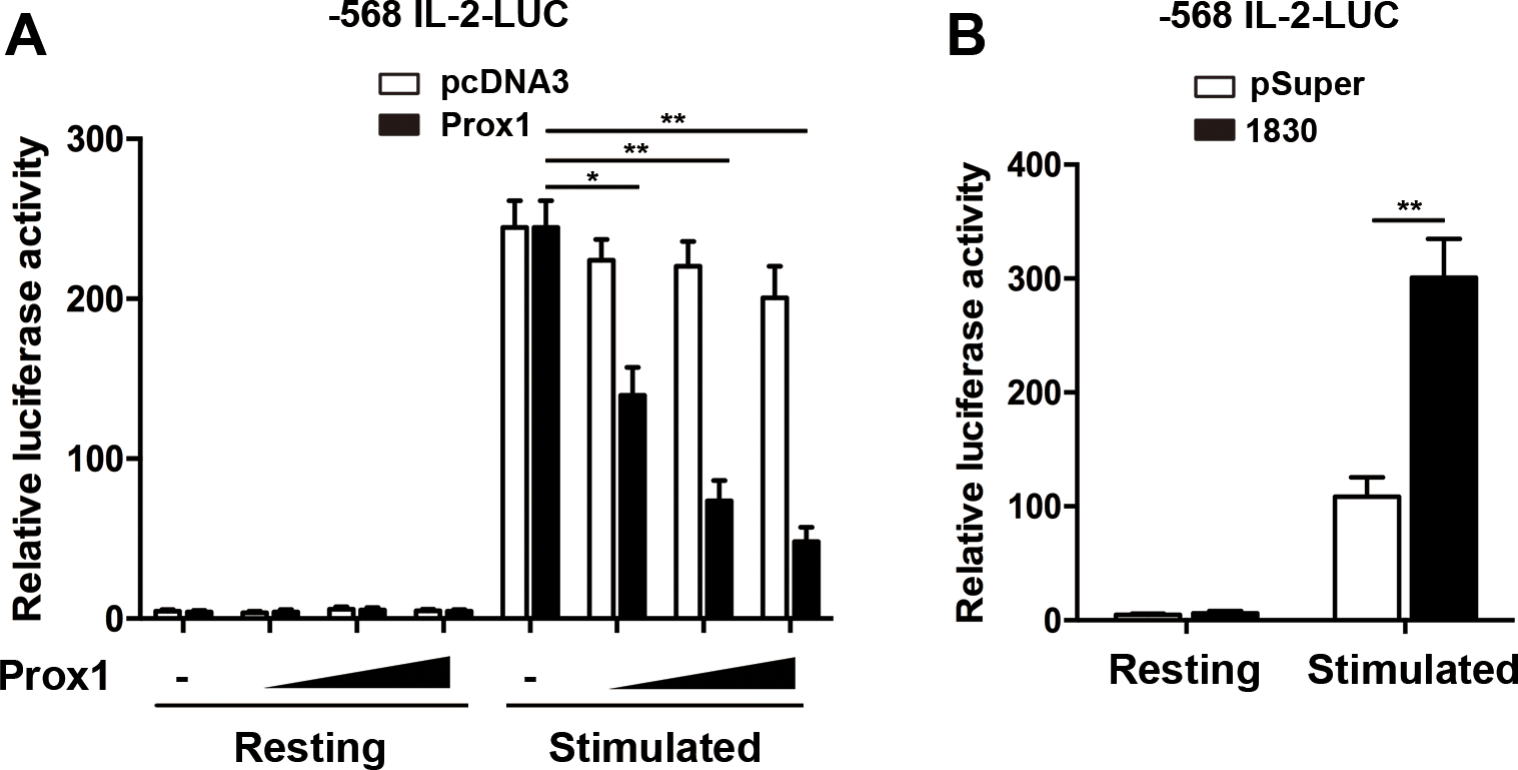
Prox1 represses IL-2 promoter activity (**A**) Jurkat cells were co-transfected with the human IL-2 promoter reporter along with increasing amounts of Prox1 plasmids (0.125, 0.25 and 0.5 μg/ml) or control vector (pcDNA3), and were stimulated with anti-CD3/CD28 Dynabeads for 24 h. (**B**) Jurkat cells were co-transfected with IL-2 promoter reporter along with Prox1-targeting siRNA, and were stimulated with anti-CD3/CD28 Dynabeads for 24 h. Relative luciferase activities were measured with dual luciferase assay, and the luciferase reporter gene was normalized with a renilla vector. The data from represent the mean ± SEM of three experiments, *P < 0.05, **P < 0.01, one-way ANOVA (A), Student’s *t*-test (B).

To examine whether Prox1 inhibited IL-2 expression by interacting with its promoter, we employed the luciferase reporter, comprising the IL-2 promoter region (–568 bp). We transfected Jurkat cells with the luciferase reporter together with Prox1 or control plasmid. The stimulation with anti-CD3/CD28 Dynabeads enhanced the luciferase activity driven by IL-2 promoter, whereas overexpression of Prox1 inhibited the luciferase activity (Figure 4A). The role of Prox1 in regulation of IL-2 transcription was further examined when endogenous Prox1 was silenced. The Prox1 knockdown augmented IL-2-luciferase activity (Figure 4B).

### Prox1 is associated with the minimal IL-2 promoter

**Figure 5:**
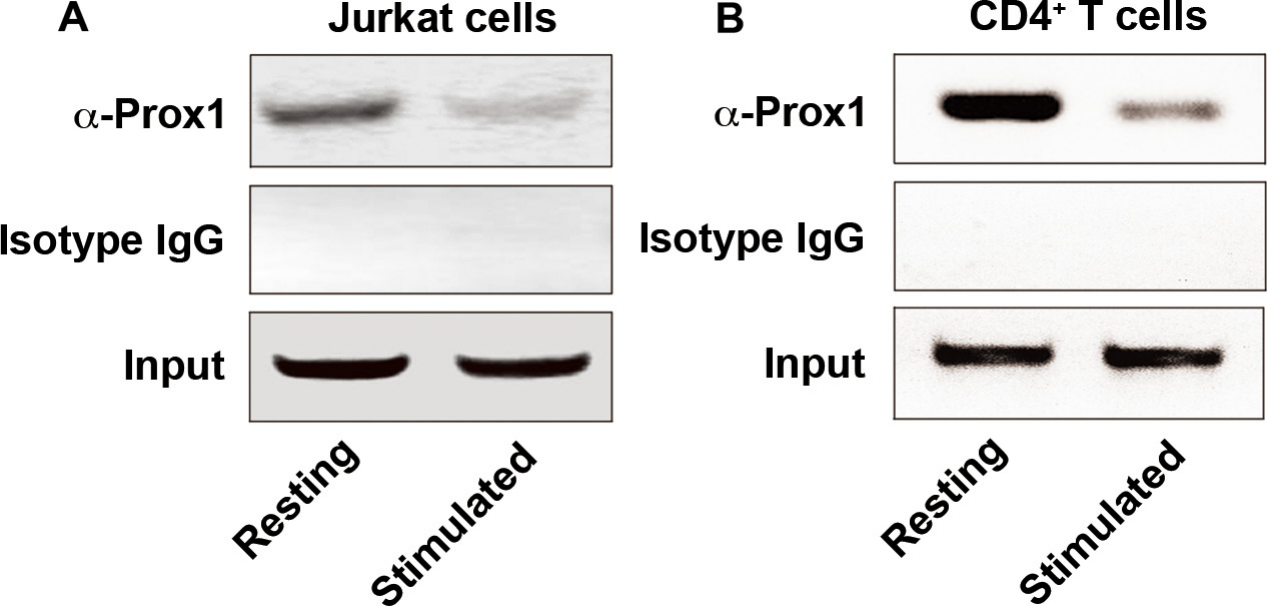
Prox1 is associated with IL-2 promoter Chromatin was extracted from (**A**) Jurkat cells and (**B**) naïve CD4^+^ T cells unstimulated or stimulated with anti-CD3/CD28 Dynabeads for 24 h, and precipitated with anti-Prox1 antibody or isotype IgG. The DNA sequence containing minimal IL-2 promoter (–256 to -46, product size: 211 bp) was analyzed by PCR. Data represent one of three separate experiments.

Furthermore, we investigated whether endogenous Prox1 was associated with the IL-2 promoter by conducting ChIP assays. Immunoprecipitation of the crosslinked chromatin from Jurkat cells or naïve CD4^+^ T cells with anti-Prox1 antibodies, but not with isotype IgG, enriched significantly the minimal IL-2 promoter within a region between -256 and -46 (Figure 5A and 5B), but not in the genomic region located between -2000 and -1820 upstream of IL-2 mRNA start site, or β-actin promoter (data not shown). The binding between Prox1 and IL-2 promoter was significantly decreased in cells treated with anti-CD3/CD28 Dynabeads (Figure 5A and 5B). Although the homeo/prospero domain in Prox1 might have DNA-binding capacity [19], the direct binding between Prox1 and promoter sequences was only observed in rare cases [20]. Since there is no direct binding between Prox1 and IL-2 promoter sequences (data not shown), these data suggested a possibility that other proteins might be involved in the interaction between Prox1 and IL-2 promoter.

### Prox1 represses NFAT2-mediated transactivation

**Figure 6:**
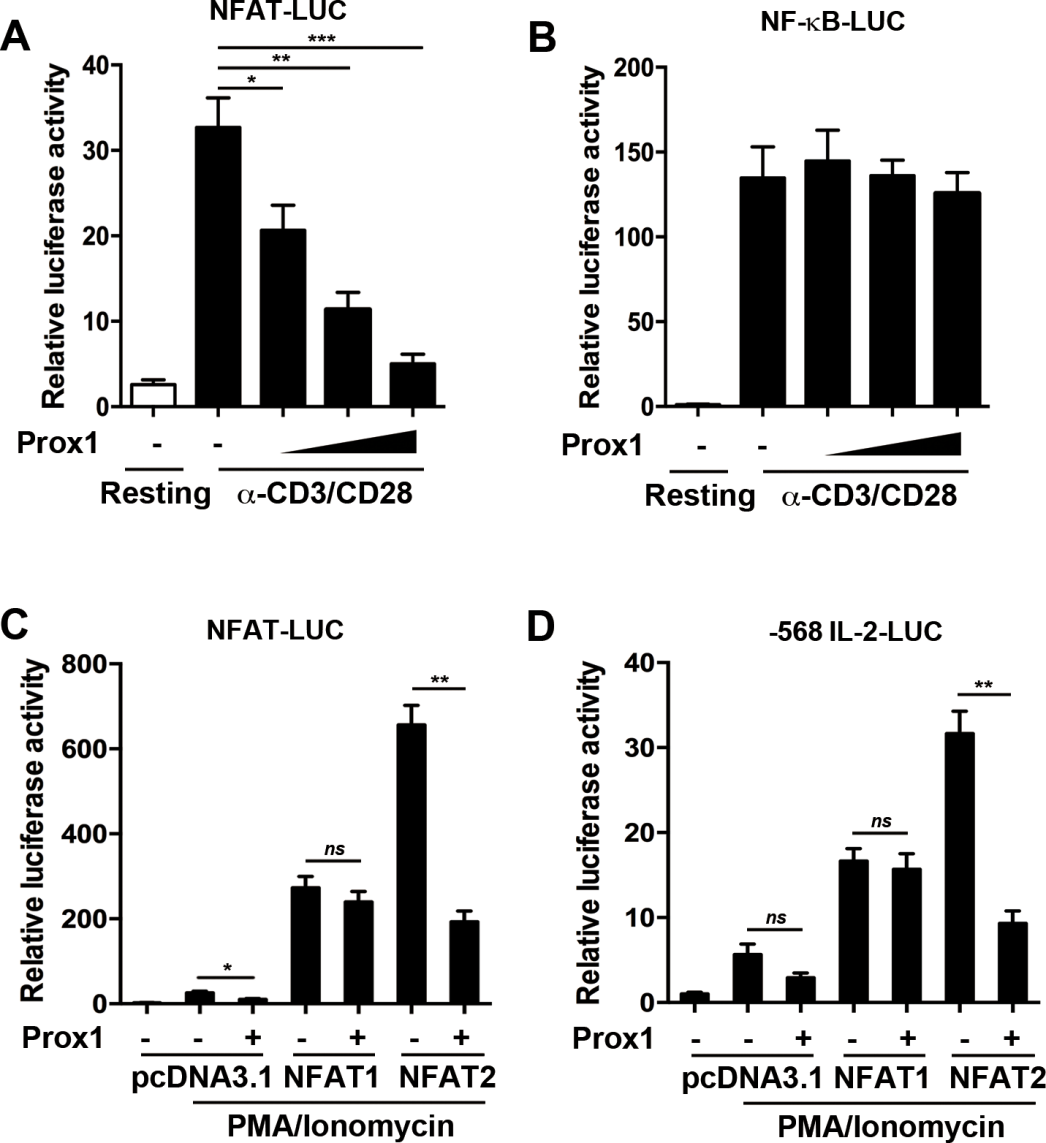
Prox1 abrogates NFAT2-mediated transactivation Jurkat cells were co-transfected with the (**A**) NFAT-luciferase reporter or (**B**) NF-κB-luciferase reporter along with the increasing amount of Prox1 plasmids (0.125, 0.25 and 0.5 μg/ml), and were stimulated with anti-CD3/CD28 Dynabeads for 24 h. Relative luciferase activities were measured by dual luciferase assay. HEK293T cells were transfected with NFAT1, NFAT2, or control vector (pcDNA3.1), Prox1 plasmids, along with (**C**) NFAT-luciferase reporter or (**D**) IL-2-luciferase reporter. The renilla vector as an internal control was used. The cells were stimulated with PMA/ionomycin for 24 h, and relative luciferase activities were analyzed by dual luciferase assay. The data from represent the mean ± SEM of three experiments, *P < 0.05, **P < 0.01, ***P < 0.001, one-way ANOVA (A and B), Student’s *t*-test (C and D); *ns*, not significant.

NFAT has been identified as one of the major transcription factors inducing IL-2 expression in activated T cells [21]. Previous studies have shown that Prox1 acted as a co-repressor for multiple DNA-binding factors [13, 18, 22, 23]. This led us to speculate that Prox1 might function as a co-repressor of NFAT. To test this speculation, we used a luciferase reporter driven by NFAT element of the promoter of IL-2. The overexpressed Prox1 significantly inhibited the luciferase activity (Figure 6A). We further investigated whether Prox1 suppressed NF-κB-mediated transcription using a luciferase reporter containing a responsive NF-κB element. As shown in Figure 6B, Prox1 did not repress NF-κB transactivation.

NFAT1 and NFAT2 are the main NFAT family members in peripheral T cells [24]. Since there is no functional expression of NFAT1, NFAT2 and Prox1 in HEK293T cells, we transfected HEK293T cells with NFAT1 or NFAT2, along with Prox1 plasmids, and searched for the primary target of Prox1. PMA/ionomycin was used to induce the activation of NFAT. As shown in Figure 6C, without ectopic NFAT1 or NFAT2, the NFAT-luciferase activity was slightly increased by PMA/ionomycin. Ectopic NFAT1 enhanced the luciferase activity, but its activity did not change in the presence of Prox1. NFAT2 similarly increased the NFAT-luciferase activity but Prox1 markedly diminished NFAT2-mediated stimulation of the luciferase activity (Figure 6C).

Next, a IL-2 promoter-driven luciferase reporter was adopted to confirm the result. PMA/ionomycin upregulated the IL-2-luciferase activity in the presence of NFAT1 or NFAT2 (Figure 6D). Prox1 repressed the NFAT2-dependent IL-2-luciferase activity, while NFAT1-enhanced promoter activity was not significantly decreased (Figure 6D). Taken together, these results suggested that Prox1 interfered and inhibited the transactivation ability of NFAT2.

### Prox1 is physically associated with NFAT2

**Figure 7:**
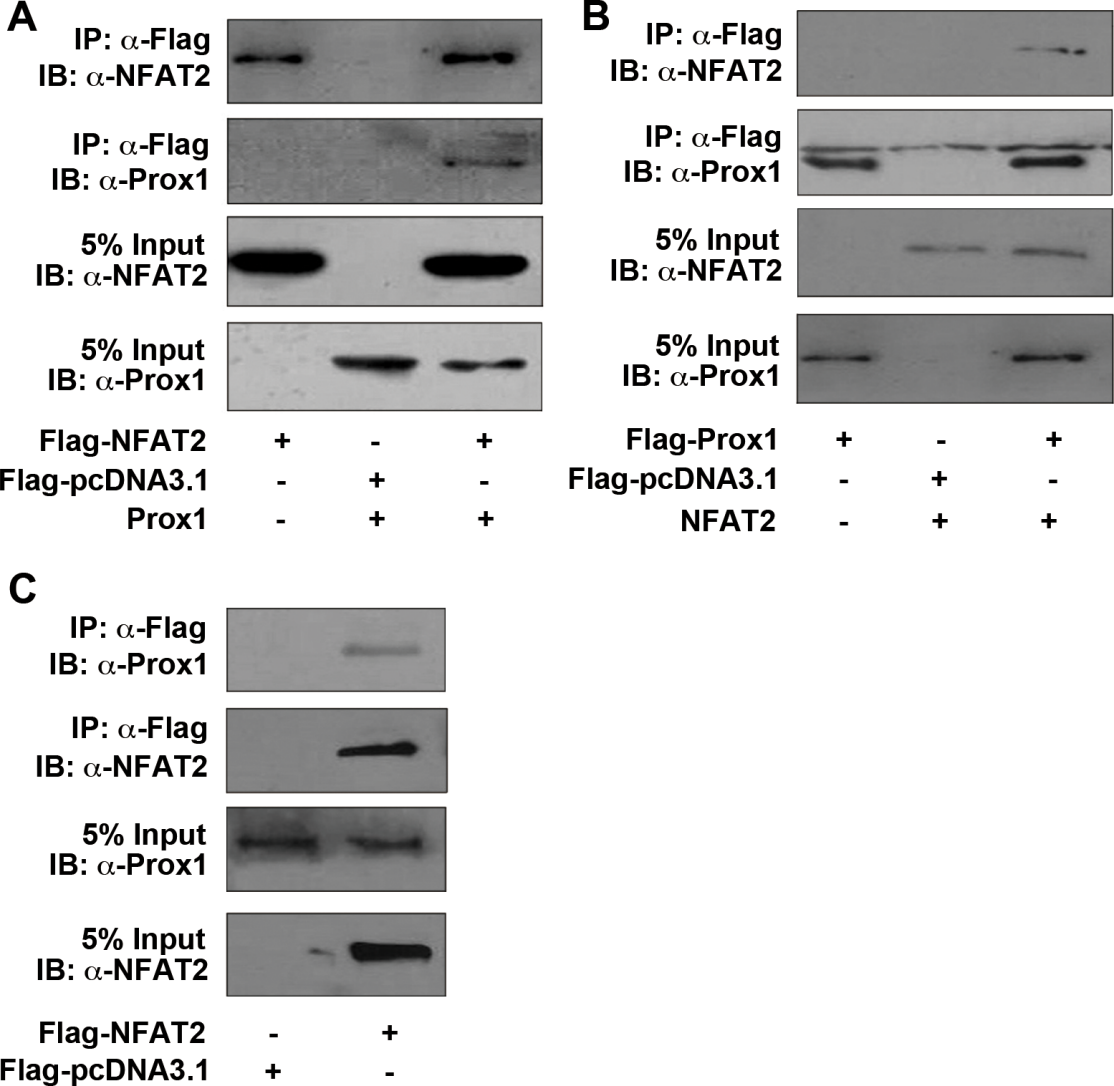
Prox1 is physically associated with NFAT2 (**A**) HEK293T cells were transiently transfected with Prox1, along with Flag-NFAT2 or control vector (Flag-pcDNA3.1) and immunoprecipitated using an anti-Flag antibody. Immunoblotting of cell extracts before immunoprecipitation (5% Input), as well as the immunoprecipitates was performed with anti-NFAT2 or anti-Prox1 antibodies. (**B**) HEK293T cells were transiently transfected with NFAT2, along with Flag-Prox1 or control vector (Flag-pcDNA3.1) and immunoprecipitated by using an anti-Flag antibody. Immunoblotting of cell extracts before immunoprecipitation (5% Input), as well as the immunoprecipitates was performed with anti-NFAT2 or anti-Prox1 antibodies. (**C**) Huh7 cells were transiently transfected with Flag-NFAT2 or control vector (Flag-pcDNA3.1) and immunoprecipitated with an anti-Flag antibody. Immunoblotting of cell extracts before immunoprecipitation (5% Input), as well as the immunoprecipitates was performed with anti-NFAT2 or anti-Prox1 antibodies. All results are representative of at least three independent experiments. IP, immunoprecipitation; IB, immunoblot.

Next, we investigated whether Prox1 could physically interact with NFAT2. Prox1, along with Flag-tagged NFAT2, were overexpressed in HEK293T cells. We showed that Prox1 physically interacted with the NFAT2 by co-IP (Figure 7A). The co-IP analysis was also carried out in the inverse manner by overexpressing NFAT2 and Flag-tagged Prox1. Immunoprecipitation with anti-Flag antibody followed by immunoblotting with anti-NFAT2 antibody, supported the interaction between Prox1 and NFAT2 (Figure 7B).

As HEK293T cells lack endogenous Prox1 expression, we further examined whether endogenous Prox1 in Huh7 cells was also associated with NFAT2. We transfected Huh7 cells with a Flag-tagged NFAT2, and demonstrated that endogenous Prox1 was also associated with NFAT2 using co-IP (Figure 7C).

### The N-terminal region of Prox1 directly interacts with NFAT2

**Figure 8:**
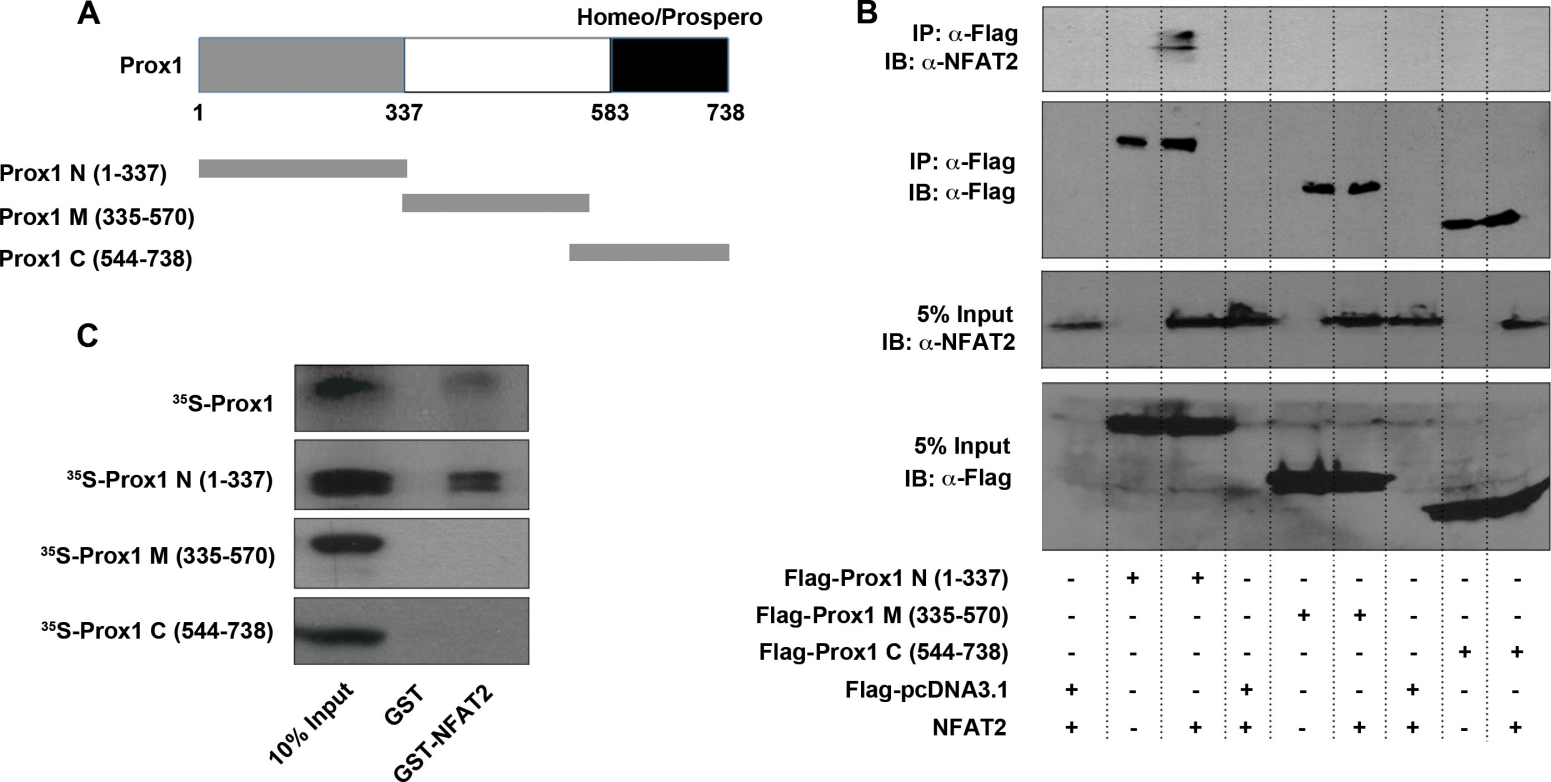
N-terminal region of Prox1 directly interacts with NFAT2 (**A**) Schematic representation of Prox1 domain organization and the truncated Prox1 proteins used in following experiments. (**B**) HEK293T cells were transiently transfected with NFAT2, along with Flag-Prox1 N (1-337), M (335-570) and C (544-738), or control vector (Flag-pcDNA3.1), and immunoprecipitated by using an anti-Flag antibody. Immunoblotting of cell extracts before immunoprecipitation (5% Input), as well as the immunoprecipitates was performed with anti-NFAT2 and anti-Flag antibodies. IP, immunoprecipitation; IB, immunoblot. (**C**) GST-fused NFAT2 protein was expressed in *E. coli* BL21 and purified using Glutathione-Sepharose beads. Purified GST-NFAT2 protein or GST alone (negative control) bound to the beads was incubated with ^35^S-labeled Prox1 and a series of ^35^S-Prox1 fragments. The reactions were analyzed by Western blot, and bound proteins were visualized by autoradiography. The input represents 10% of the labeled proteins used for the pull-down assay. All results are representative of at least three independent experiments. IP, immunoprecipitation; IB, immunoblot.

To determine which regions of Prox1 were responsible for its interaction with NFAT2, deletion mutants of Prox1 were generated as previously described [14, 18]. HEK293T cells were transfected with NFAT2, along with different Flag-tagged Prox1 fragments [N (1–337), M (335–570) and C (544–738)] (Figure 8A). Co-IP analysis with an anti-Flag antibody showed that NFAT2 co-immunoprecipitated with Prox1 N (1–337), but not with Prox1 M (335–570) and C (544–738) (Figure 8B).

To further verify the interaction between NFAT2 and Prox1 fragments, GST pull-down assays were performed. Both full-length Prox1 and the Prox1 N (1–337) displayed positive binding of NFAT2, whereas the Prox1 M (335–570) and C (544–738) showed no binding of NFAT2 at all (Figure 8C). These results suggested that Prox1 directly interacted with NFAT2 mainly through its N-terminal (1–337) region.

### Prox1 N-terminal region represses IL-2 expression

**Figure 9:**
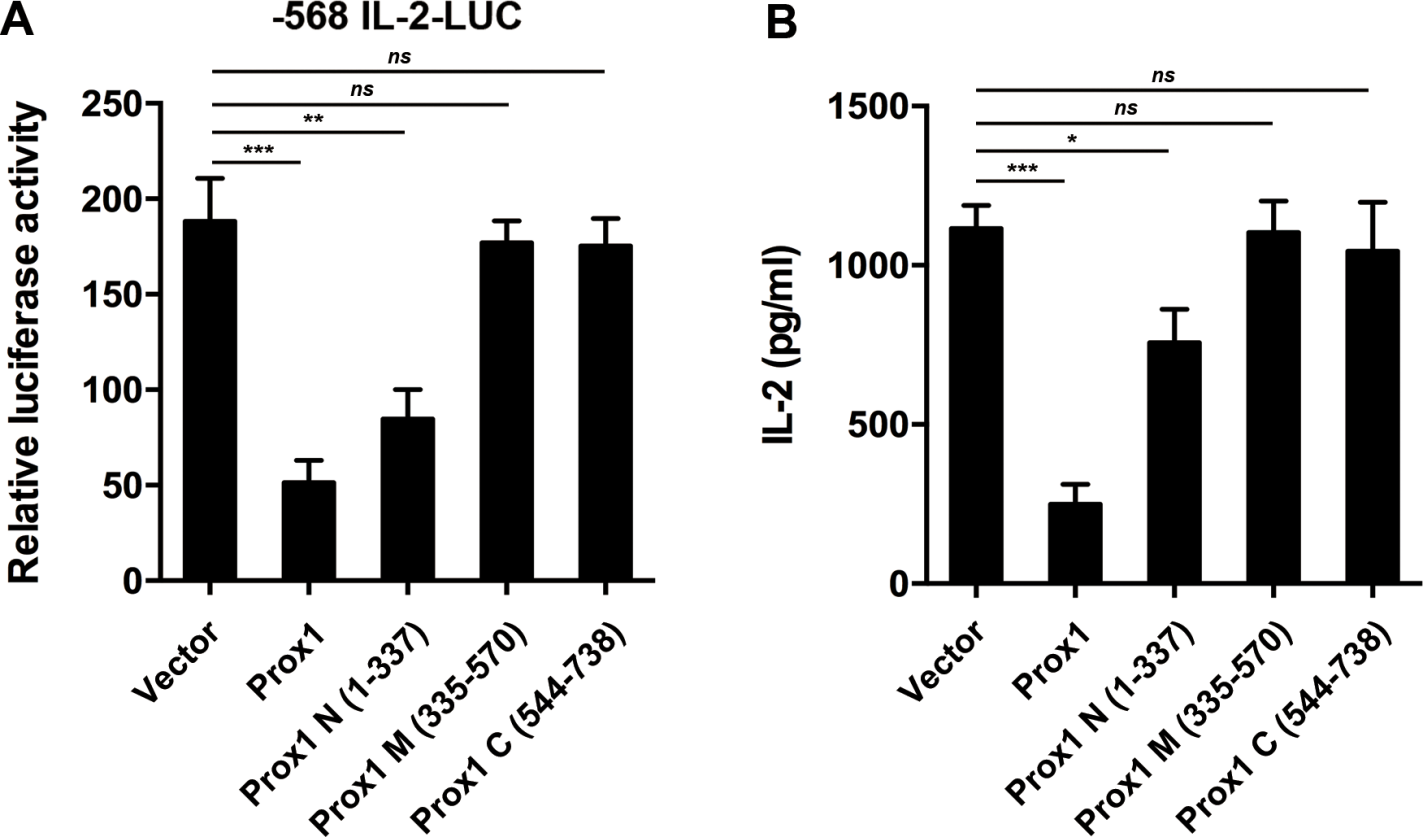
N-terminal region of Prox1 repressed IL-2 expression (**A**) Jurkat cells were co-transfected with the human IL-2 promoter reporter along with Prox1 as well as indicated Prox1 fragments, and were stimulated with anti-CD3/CD28 Dynabeads for 24 h. Relative luciferase activities were measured with dual luciferase assay. The luciferase reporter gene was normalized with a renilla vector. (**B**) Jurkat cells were transfected with Prox1 as well as indicated Prox1 fragments, and were stimulated with anti-CD3/CD28 Dynabeads for 24 h. IL-2 protein levels were measured by ELISA. The data from represent the mean ± SEM of three experiments, *P < 0.05, **P < 0.01, ***P < 0.001, one-way ANOVA.

To investigate the functional impact of Prox1-NFAT2 interaction, we transfected Jurkat cells with Prox1 mutants, and then examined L-2 promoter activity and IL-2 production. Full-length Prox1 and Prox1 N (1–337), which displayed strong binding for NFAT2, repressed the activity of IL-2 promoter (Figure 9A) and secretion of IL-2 (Figure 9B), whereas Prox1 M (335–570) and C (544–738), showed no repression corresponding to their negative binding to NFAT2 (Figure 9A and 9B). Thus, the repressive effect of Prox1 was dependent upon the interaction between NFAT2 and N-terminus of Prox1.

## DISCUSSION

IL-2 is the key cytokine in the initiation and maintenance of T cell response, and modulation of IL-2 expression can alter the outcome of autoimmune diseases [25]. Previous studies have suggested that several transcription factors were decreased in the process of T cell stimulation, and thus might serve as negative regulators [26–28]. In the present study, we described a new molecular function of Prox1, as well as the identification of IL-2 as a target gene regulated by Prox1. Specifically, we demonstrated through several lines of evidence that Prox1 functioned as a transcriptional repressor in IL-2 gene expression: (1) we found that T cell activation downregulated Prox1 expression; (2) Prox1 repressed IL-2 promoter activation; (3) in addition, we demonstrated that Prox1 blocked the ability of the key transcription factor NFAT2 to transactivate IL-2 gene; (4) we proved that Prox1 was physically associated with NFAT2; (5) N-terminal region of Prox1 might account for the interaction and repressive effect.

Given its central importance as T cell growth factor [29], IL-2 expression has multiple levels of lineage-specific transcriptional control, including DNA methylation, chromatin remodeling, and interaction of cis- and trans-acting transcription factors [2]. An interacting network of transcription factors co-regulates the rate of IL-2 transcription, such as NFAT family proteins, AP-1, NF-κB, and OCT-1. The 300 bp upstream of the transcription start site of IL-2 gene has been the focus of previous studies on transcriptional regulation [29]. The binding of Prox1 with the IL-2 promoter in unstimulated T cells suggested that Prox1 might serve as a safeguard molecule in preventing the aberrant activation of IL-2.

The target promoters are regulated not only by DNA-binding transcription factors but also by other bridging molecules positively or negatively. In previous studies, Prox1, as a co-regulator, interacted with several transcription factors such as LRH1, HNF4α, LSD1 and PPAR γ [13, 14, 18, 30]. While NFAT proteins are master regulators of IL-2 transcription, several NFAT partners are able to interact with NFAT resulting in synergistic activation or repression of the process [21]. Thus, we speculated that NFAT might be another target for Prox1-mediated repression. In the present study, NFAT2, but not NFAT1-mediated transactivation was suppressed by Prox1. Of the 5 NFAT family members (NFAT 1–5), NFAT1 and NFAT2 are the major molecules in peripheral T cells. NFAT1 is constitutively expressed in T cells, whereas the expression level of NFAT2 is markedly upregulated following T cell stimulation. Previous studies using gene-knockout mice suggested that there is some functional redundancy in different NFAT molecules [31]. It is only when more than one NFAT member has been knocked out that changes in functions of immune system become significant [31]. However, these data also demonstrated that certain NFAT molecules specifically regulated different T cell functions. It was previously postulated that NFAT family members might differ in their ability to interact with co-regulators, and the present data further supported this theory. Moreover, NFAT2 binding sites were found within interleukin-17A (IL-17A) promoter, and expression of IL-17A in human CD4^+^ T cells was augmented by the introduction of NFAT2 [32, 33]. Further studies would be required to examine the expression kinetics of Prox1 during Th17 cell differentiation, and it is possible that NFAT2-transactivated IL-17A expression would also be interfered by Prox1.

Although Prox1 interacts with several DNA-binding factors, there are clear differences in the manner by which they interact. The N-terminal region of Prox1 is capable of binding HNF4α and LRH1, while interaction of Prox1 with proliferating cell nuclear antigen (PCNA) is dependent on C-terminal region [22, 30, 34]. We recently reported that both the N-terminus and the C-terminus of Prox1 are capable of binding LSD1 [14]. In our study, co-IP and GST pull-down analysis with different Prox1 segments indicated that the Prox1 N-terminus, but not the C-terminal homeo/prospero domain, was capable of binding NFAT2.

Functions of proteins can be achieved by post-translational modification, for example with small ubiquitin-related modifier (SUMO) [35, 36]. We previously demonstrated that the function of Prox1 as a co-repressor might be inhibited by sumoylation [37]. It would be interesting to investigate whether sumoylation interferes with Prox1-NFAT2 interaction.

Physiologically, a major question in immunology, is the molecular mechanism that underlies the activation and transition of naïve T helper cells to the status of proliferating T helper precursors, and then to the various T helper lineages. The downregulation of Prox1 along with the repressive effects of Prox1 on IL-2 expression indicated that endogenous Prox1, may be an important regulator in the activation of resting T cells *in vivo*. Such mechanisms possibly protect gene expression from aberrant induction, which leads to uncontrolled and disastrous immune responses. Several questions remain, including how expression of Prox1 is downregulated during T cell activation; whether Prox1-NFAT2 interaction affects the capability of NFAT2 to bind IL-2 promoter; and which other co-repressors are recruited by Prox1 to the promoters of NFAT2-responsive genes.

In summary, we have shown that Prox1 can inhibit NFAT-driven of IL-2 expression in CD4^+^ T cells through direct interacting with NFAT2 and repressing the transactivation activity of NFAT2. The Prox1 N-terminal domain is responsible for the interaction, as well as repression. Our study has suggested that Prox1 acts as a fine-tuning mechanism of T cell immune responses by negatively modulating IL-2 expression. Future investigations should also explore the possibility of Prox1 as a potential target for therapeutic intervention of immunological disorders.

## MATERIALS AND METHODS

### Cells and stimulation

The study was approved by the ethics committee of Fudan University, and was carried out in accordance with the ethical principles in the Declaration of Helsinki. Peripheral blood samples (10–30 ml) from 6 healthy adults (faculty of Key Laboratory of Medical Molecular Virology, Fudan University, Shanghai, China) were collected in heparinized tubes. For the isolation of peripheral blood mononuclear cells (PBMCs), the anticoagulated blood was mixed with an equal volume of PBS. The diluted blood was slowly layered over Histopaque-1077 (Sigma, St. Louis, MO, USA) by gently pipetting the diluted blood down the side of the tube, and was then centrifuged for 20 min at 800 g at 22°C, with no brake. The mononuclear cell layer was transferred into 10 ml PBS and centrifuged for 10 min at 400 g at 4°C. After two washings with RPMI 1640 (Invitrogen, Carlsbad, CA, USA), PBMCs were adjusted to 1 × 10^6^ cells/ml in RPMI 1640 and preserved at 37°C under 5% CO_2_ until used for experiments or further T cell isolation. For PBMCs stimulation, PBMCs were treated with 10 μg/ml phytohemagglutinin-L (PHA-L; Sigma) for 24 h. For the isolation of human naïve CD4^+^ T cells, CD4^+^ T cells were first isolated from PBMCs by depletion of non-CD4^+^ T cells using a CD4^+^ T Cell Isolation Kit II (Miltenyi Biotec, Bergisch Gladbach, Germany). Then the naïve CD4^+^ T cells were isolated using a Naïve CD4^+^ T cell Isolation Kit (Miltenyi Biotec). Jurkat cells were cultured in RPMI 1640 supplemented with 10% fetal bovine serum (Invitrogen), penicillin (50 units/mL), and streptomycin (50 mg/mL) at 37°C under 5% CO_2_. Naïve CD4^+^ T cells or Jurkat cells were stimulated with Dynabeads Human T-activator CD3/CD28 (Invitrogen) according to the manufacturer’s instructions. These superparamagnetic beads combined with CD3 and CD28 antibodies provided primary and co-stimulatory signals, necessary for the activation of T cells, without requiring antigens or APCs. Embryo kidney cell line HEK293T and human hepatoma cell line Huh7 were maintained in Dulbecco’s modified Eagle medium (DMEM) (Invitrogen) supplemented with 10% fetal bovine serum, and cultured at 37°C with 5% CO_2_.

### RT-PCR and real-time PCR

Total RNA was extracted from cells using RNeasy Mini Kit (Qiagen, Valencia, CA, USA) in accordance with the manufacturer’s instructions. A total of 1 μg total RNA isolated was reverse transcribed with the reverse transcriptase kit (Takara, Shiga, Japan) according to the manufacturer’s protocol. Prox1, IL-2 and GAPDH were amplified using ExTaq DNA polymerase (Takara). Polymerase chain reaction assay cycles were as follows: 94°C for 5 min, 35 cycles of 94°C for 30 sec, 56°C for 30 sec, and 72°C for 30 sec. The PCR products were visualized on 2% agarose gels and ethidium bromide staining. To check the mRNA levels of Prox1, IL-2 and GAPDH, real-time PCR was performed in triplicate with a real-time PCR system (ABI PRISM 7500; Applied Biosystems, Foster City, CA, USA) using a SYBR detection kit (Takara) according to the standard protocol. The mRNA levels of each target gene were normalized to the levels of GAPDH and were represented as fold induction. The following primer sets were adopted (forward/reverse). Prox1: 5′-AAC TAG GGA TAC CAC GAG TC-3′/5′-CTT CAC TAT CCA GCT TGC AG-3′; IL-2: 5′-ACC TCA ACT CCT GCC ACA AT-3′/5′-GCA CTT CCT CCA GAG GTT TG-3′; GAPDH: 5′-AAT CCC ATC ACC ATC TTC C-3′/5′-TTG AGG CTG TTG TCA TAC TTC T-3′.

### Western blot

After treatment, cells were harvested and lysed with RIPA buffer (Shenggong, Shanghai, China). Cytosol proteins from cells (50 μg/lane) were separated by electrophoresis in 10% SDS-polyacrylamide gel and then transferred onto a PVDF membrane (Millipore, Bedford, MA, USA). Subsequently, the membrane was blocked in 2% BSA for 1 h at room temperature and incubated overnight at 4°C with the primary antibodies against Prox1 (Upstate, Lake Placid, NY, USA) and β-actin (Sigma). After washing with PBST (phosphate buffer solution containing Tween-20) five times, the membranes were probed with horseradish peroxidase (HRP)-conjugated secondary antibodies. The membranes were developed with an enhanced chemiluminescence system from Amersham and exposed to X-ray film (Fuji Photo Film, Shanghai, China).

### Plasmid constructs

The human Prox1 full-length cDNA was previously cloned in the pcDNA3 vector [13, 14]. The pcDNA3.1-Flag (N-terminal) vector was a gift from Dr. Chen Wang (Institute of Biochemistry and Cell Biology, Chinese Academy of Sciences). Full-length Prox1, as well as Prox1 N (1–337), M (335–570) and C (544–738) cDNA were amplified by PCR and inserted into the BamHI and EcoRI sites of the pcDNA3.1-Flag vector to generate the pcDNA3.1-Flag-Prox1, pcDNA3.1-Flag-Prox1 N (1–337), pcDNA3.1-Flag-Prox1 M (335–570) and pcDNA3.1-Flag-Prox1 C (544–738) respectively. The pRSV-NFAT2 plasmid containing human NFAT2 full-length cDNA was provided by Dr. Anjana Rao (Harvard Medical School). The NFAT2 cDNA was subcloned into the BamHI and EcoRV sites of the pcDNA3.1 vector (Invitrogen) to generate the pcDNA3.1-NFAT2 vector. The NFAT2 cDNA was also subcloned into the ClaI and EcoRV sites of the pcDNA3.1-Flag vector to obtain the pcDNA3.1-Flag-NFAT2. The Prox1 full-length cDNA was cloned from pcDNA3-Prox1 into the lentiviral vector pWPI.1 (Addgene, Cambridge, MA, USA) to achieve overexpression as previously described [14]. For the production of GST-NFAT2 fusion proteins, the NFAT2 full-length cDNA was amplified and inserted into the BamHI and EcoRI sites of pGEX-4T-1 vector (GE Healthcare, Piscataway, NJ, USA). The pIL2-568 plasmid containing human IL-2 promoter (-568 to +50) was a gift from Dr. Christopher B. Wilson (University of Washington) [38]. The IL-2 promoter (-568 to +50) was subcloned into pGL3-basic (Promega, Madison, MI, USA). pcDNA3.1-NFAT1 vectors and NFAT-luciferase reporters were kindly provided by Dr. Lin Li (Institute of Biochemistry and Cell Biology, Chinese Academy of Sciences). NF-κB-luciferase reporter was obtained from Promega.

### Lentivirus infection

Helper plasmids pSPAX2 (Addgene) and pMD2.G (Addgene) were co-transfected with the pWPI.1 vector co-expressing green fluorescent protein (GFP) and Prox1 into HEK293T cells to package recombinant lentiviruses using polyethylenimine (PEI, Sigma). Lentivirus-containing supernatant was collected after 72 h of incubation with the transfected packaging cells. Jurkat cells were infected by resuspending cells in lentivirus-containing supernatant. After 48 h of incubation at 37°C, the GFP^+^ cells were purified by a fluorescence-activated cell sorter (BD FACSAria II, BD Bioscience), and were used for subsequent treatment.

### Small interfering RNA (siRNA)

For RNA interference of Prox1, coding sequences for Prox1-1830 small interfering RNA (5′-AGT TCA ACA GAT GCA TTA C-3′) were inserted in hairpin format into pSuper vector (OligoEngine, Seattle, WA, USA) as previously described [18, 37]. Jurkat cells were transfected with either pSuper or pSuper-Prox1-1830 using Lipofectamine 2000 (Invitrogen). After 36 h of incubation at 37°C, the GFP^+^ cells were purified by the fluorescence-activated cell sorter, and were used for subsequent treatment.

### IL-2 ELISA

The quantitative measurement of IL-2 secreted from treated Jurkat cells in culture supernatant was carried out with an ELISA kit (R&D Systems, Minneapolis, MN, USA) according to the manufacturer’s instruction.

### Reporter assays

For reporter assays, a luciferase reporter plasmid and an expression plasmid were co-transfected using Lipofectamine 2000, along with pRL-SV40, an internal control vector containing the renilla luciferase gene (Promega), in a ratio of 50:1, in order to normalize the transfection efficiency. 24 h after transfection, the cells were treated with anti-CD3/CD28 Dynabeads or PMA (25 ng/ml, Sigma) and ionomycin (1 μM, Calbiochem, San Diego, CA, USA) for 24 h. After the treatment, cells were gently rinsed with PBS and harvested with Passive Lysis Buffer (Promega). The Dual luciferase Reporter Assay System (Promega) was used to measure luciferase activity. 20 μl of cell lysate were added to 40 μl of luciferase assay reagent II (Promega) and the firefly luminescence was read using a Modulus 96 Luminometer. Next, 40 μl of Stop & Glo reagent (Promega) was added to the lysates and renilla luminescence was read. Luminescence values of firefly were normalized with renilla for each construct within an experiment.

### Chromatin immunoprecipitation (ChIP)

ChIP assays were performed following a published protocol [39]. Briefly, chromatins were sheared by sonication. Precleared extracts were immunoprecipitated with rabbit anti-Prox1 antibody (Upstate) or rabbit IgG (Sigma) at 4°C overnight. DNA was isolated from precipitated complexes and analyzed by PCR using primers for the minimal human IL-2 promoter (–256 to –46, product size: 211 bp): 5′-CTA CTC ACA GTA ACC TCA ACT CCT-3′/5′-TGT AGA ACT TGA AGT AGG TGC ACT-3′. An aliquot of total input nuclear extract was used as loading control.

### Co-immunoprecipitation (Co-IP)

HEK293T cells were transiently co-transfected with the expression plasmids either for Prox1 and Flag-NFAT2 or Flag-Prox1 and NFAT2 with the PEI precipitation method [40]. Huh7 cells were transiently transfected with Flag-NFAT2 with the PEI precipitation method. 48 h post-transfection, co-IP was carried out as previously described [13]. Briefly, 1 mg of protein was incubated with anti-Flag agarose affinity gel (Sigma) for 6 h at 4°C. Precipitates were separated on a 10% SDS-polyacrylamide gel and analyzed by immunoblotting. Immunoblottings of Prox1, NFAT2 or Flag-tagged Prox1 fragments were performed with anti-Prox1 antibody (Upstate), anti-NFAT2 antibody (eBioscience) and anti-Flag M2 antibody as primary antibody respectively. HRP-labeled anti-rabbit IgG (DAKO, Carpinteria, CA, USA) or anti-mouse IgG (DAKO) was used as secondary antibody.

### Glutathione S-transferase (GST) pull-down

^35^S-methionine-labeled proteins were produced *in vitro* using TNT Quick Coupled Transcription/Translation System (Promega) following the manufacturer’s instruction. GST fusion proteins were expressed in *E.coli* BL21 (pLys) (Novagen, LaJolla, CA, USA) induced with 0.5 mM isopropyl-β-D-thiogalactoside for 12 h at 24°C. Pull-down assays were performed with 2 mg of a GST fusion protein and 10 μl of a labeled protein. Purification of recombinant GST fusion proteins and GST pull-down assays were performed essentially as described previously [13].

### Statistical analysis

All results are shown as mean and the standard error of the mean (mean ± SEM). The data were assessed for normal Gaussian distribution with Kolmogorov-Smirnov test. We used two-tailed Student’s *t*-test to determine significances between two groups. We did analyses of multiple groups by one-way analysis of variance (ANOVA) with Bonferroni post hoc test. A value of *P* < 0.05 was considered significant, where *P < 0.05, **P < 0.01 and ***P < 0.001. Analyses and graphical representation were performed using GraphPad Prism 7 software (GraphPad Software, La Jolla, CA, USA).

## SUPPLEMENTARY MATERIALS FIGURE



## Abbreviations

Prox1: prospero-related homeobox 1
TCR: T cell receptor
NFAT2: nuclear factor of activated T cells 2
AP-1: activated protein-1
NF-κB: nuclear factor-κB
CBP/p300: CREB-binding protein (CBP) and p300
ICER: inducible cAMP early repressor
PPARγ: peroxisome proliferator-activated receptor γ
LRH1: liver receptor homologue 1
CYP7A1: cholesterol 7α-hydroxylase
LSD1/NuRD: lysine-specific demethylase 1/nucleosome remodeling and histone deacetylase
GST: glutathione S-transferase
