# Supplementary material for: Prox1 represses IL-2 gene expression by interacting with NFAT2
Source: Oncotarget. 2017 Apr 20;8(41):69422–34. doi: 10.18632/oncotarget.17278 (PMC5642489; doi:10.18632/oncotarget.17278)
Supplement: Supplementary file 1 [file oncotarget-08-69422-s001.pdf]

Prox1 represses IL-2 gene expression by interacting with NFAT2

Supplementary Materials

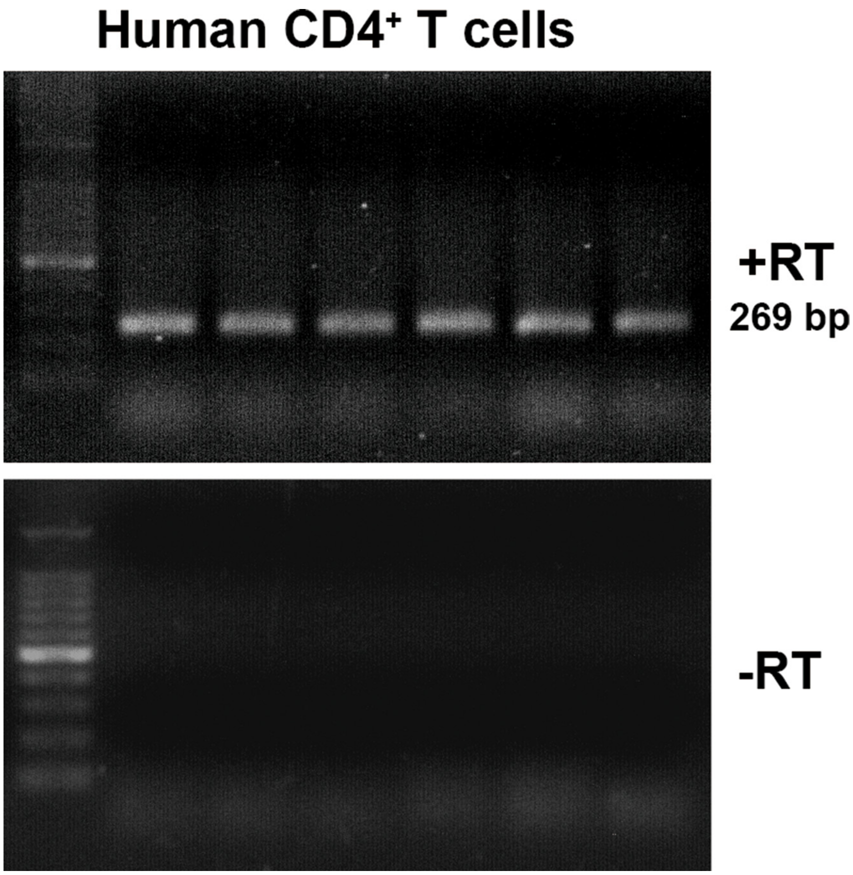

Supplementary Figure 1: Expression of Prox1 in human T cells. Naïve CD4+ T cells were isolated from six healthy donors, and then Prox1 mRNA was measured by RT-PCR.
